# Proteomic analysis of pregnancy-related proteins from pig uterus endometrium during pregnancy

**DOI:** 10.1186/1477-5956-9-41

**Published:** 2011-07-26

**Authors:** Jung-Il Chae, Jumi Kim, Seong G Lee, Young-Joo Jeon, Dong-Wook Kim, Yunjo Soh, Kang S Seo, Hak K Lee, Nag-Jin Choi, Joohyun Ryu, Sunghyun Kang, Seong-Keun Cho, Dong-Seok Lee, Hyung M Chung, and Deog-Bon Koo

**Affiliations:** 1 Department of Oral Pharmacology, School of Dentistry and Institute of Dental Bioscience, BK21 project, Chonbuk National University, Jeonju (651-756), Korea; 2 Department of Obstetrics and Gynecology, College of Medicine, Yeungnam University, Daegu 705-717, Korea; 3 CHA Bio & Diostech Co., Ltd. 606-16 Yeoksam 1 dong, Gangnam gu, Seoul 135-907, Korea; 4 Graduate School of Life Science, CHA Stem Cell Institute, College of Medicine, CHA University, 605-21 Yeoksam 1 dong, Gangnam gu, Seoul 135-907, Korea; 5 Department of Animal Science and Technology, Sunchon National University, Suncheon 540-742, Korea; 6 Genomic Informatics Center, Hankyong National University, 67 Sukjong-dong, Ansung-city, Kyongi-do, 456-749, Korea; 7 Department of Animal Science, College of Agricultural & Life Science, Chonbuk National University, Jeonju, Korea; 8 Medical Proteomics Research Center, KRIBB, Daejeon, Republic of Korea; 9 Depart. of Animal Science, College of National Resources and Life Science, Pusan National University, Miryang-si, Gyeongnam 627-706, Korea; 10 College of Natural Sciences, Kyungpook National University, Daegu 702-701, Korea; 11 Department of Biotechnology, College of Engineering Daegu University, 15 Jillyang Gyeongsan, Gyeongbuk 712-714, Korea

## Abstract

Many important molecular events associated with implantation and development occur within the female reproductive tract, especially within the uterus endometrium, during pregnancy periods. The endometrium includes the mucosal lining of the uterus, which provides a suitable site for implantation and development of a fertilized egg and fetus. To date, the molecular cascades in the uterus endometrium during pregnancy periods in pigs have not been elucidated fully. In this study, we compared the functional regulated proteins in the endometrium during pregnancy periods with those in non-pregnant conditions and investigated changes in expression patterns during pregnancy (days 40, 70, and 93) using two-dimensional gel electrophoresis (2-DE) and western blotting. The functional regulated proteins were identified and discovered from differentially expressed proteins in the uterus endometrium during pregnancy. We discovered 820 protein spots in a proteomic analysis of uterus endometrium tissues with 2-DE gels. We identified 63 of the 98 proteins regulated differentially among non-pregnant and pregnant tissues (matched and unmatched spots). Interestingly, 10 of these 63 proteins are development-, cytoskeleton- and chaperon-related proteins such as transferrin, protein DJ-1, transgelin, galectin-1, septin 2, stathmin 1, cofilin 1, fascin 1, heat shock protein (HSP) 90β and HSP 27. The specific expression patterns of these proteins in the endometrium during pregnancy were confirmed by western blotting. Our results suggest that the expressions of these genes involved in endometrium function and endometrium development from early to late gestation are associated with the regulation of endometrium development for maintaining pregnancy.

## Background

In mammalian reproduction, many important events, including the transport and final maturation of female and male gametes, fertilization, embryonic development, and transport of the embryo to the uterus, occur within the female reproductive tract, especially within the oviduct and uterus. Successful implantation and maintenance of pregnancy requires synchrony between embryonic development and the establishment of reciprocal interactions between the conceptus (embryo/fetus and associated extraembryonic membranes) and endometrium [1,2].

The endometrium is a plastic tissue in which cells undergo a variety of adaptation reactions in response to the physiological changes that occur in the different phases of the cycle and during embryo implantation. The endometrium is composed of three histologically distinct layers: stratum basalis (deepest layer), stratum spongiosum (intermediate layer) and stratum compactum (thinner, most superficial layer) [3]. Unlike most normal adult tissues, the functional layer of the uterine endometrium undergoes cyclical growth and tissue remodeling throughout the reproductive years. This remodeling process of endometrial tissue is regulated by several factors, such as the ovarian steroids, various cytokines and growth factors, which influence endometrial differentiation and function, pregnancy recognition signaling, uterine receptivity for blastocyst implantation, and conceptus-uterine interactions. Tissue remodeling shares features with the repair of mucosal injury, characterized by a migratory phenotype with specialized cytoskeletal and matrix-receptor reorganizations and specialized matrix-dependent signaling patterns [4-6].

Human implantation begins when the blastocyst assumes a fixed position in the uterus and establishes a more intimate relationship with the endometrium. For this relationship to be established, an ordered succession of events must occur [7,8]. However, investigation of the events occurring after implantation to maintain pregnancy in humans at the molecular level is difficult because of challenges in obtaining human tissue. Therefore, animal models are needed for studying both the molecular and the mechanical events associated with implantation and pregnancy.

As these reasons, many kinds of animals such as procine, cow or sheep were applied in reproduction research part. The pig maintain pregnant 116 days and establishment of pregnancy involves synchronization of progesterone stimulated endometrial function, blastocyst development and steroid synthetic capability. Pig implantation and placentation differ from that in both rodents and sheep because pigs have a true epitheliochorial placenta in which uterine luminal epithelium (LE) is intact throughout pregnancy. Uterine endometrial functions during the periimplantation period of pregnancy in pig are uniquely regulated through interacting effects of P4 from the corpus luteum (CL) and estrogens from the conceptus, with estrogen (E2) being the pregnancy recognition signal that redirects prostaglandin F2_ secretion from an endocrine to an exocrine mode during which it is sequestered and metabolized to prevent luteolysis of the CL.

Therefore, pigs provide a valuable comparative model to analyze implantation/placentation-associated gene and protein regulation. Although, there is still no database bearing up-to-date candidate genes and proteins for reproduction traits of pig, based on genetic similarity between human and pig and the intensive studies on human reproductive mechanism, porcine model was very valuably applied in research field about reproduction.

In various species, to maintain pregnancy, several genes are expressed and functionally activated in the uterus, especially in the endometrium [7,9]. During implantation, endometrial focal adhesions develop as aggregates composed of ECM proteins, integrins and cytoskeletal proteins, which promote and stabilize the attachment of trophectoderm [10-12]. Of all the cell adhesion molecules, endothelial markers and integrins have been extensively investigated in the endometrium and deciduas [13], but comparison of the overall protein expression profile in pregnant and non-pregnant endometria has not been adequately explored. To date, the development of a test for endometriosis has mostly concentrated on the levels of cytokines and growth factors involved in inflammation, angioneogenesis and tissues remodeling that are present in the serum, peritoneal fluid, endometrium and endometriotic lesions during pregnancy [6,14-17]. Because changes in the whole protein expression profile occur during pregnancy, proteomic techniques are now being employed to identify proteins expressed during different stages of pregnancy.

Studies on comparative transcriptomes of the human endometrium in different phases of the menstrual cycle have demonstrated differential expression of several genes regulating intracellular signaling, transcription, and metabolism in the midsecretory or receptive phase [13,18,19]. However, consensus on the number, identity, and expression pattern of the factors associated with endometrial receptivity has yet to be achieved. Moreover, microarrays do not reveal the effect of posttranscriptional or posttranslational regulation on protein expression [20]. Proteomics allows the global study of protein expression and regulation in a biological system. Proteome analysis is now widely accepted as a complementary technology to genetic profiling and is being increasingly employed in medical research to identify proteins as potential biomarkers of various disease states. Its use is also important in the quest to develop diagnostic tests for disease and to improve the understanding of specific pathways and their relationship to disease formation or development [15,21,22].

Although the validation of findings related to a single protein or small groups of proteins differentially expressed in the disease state is difficult, proteomic profiling using mass spectrometry in combination with sophisticated bioinformatics software to identify protein patterns may be able to make a significant clinical diagnostic contribution [10,23].

In a several previous reports, the finding of implantation factors in animal models had clinical significance. In all animal models of implantation, the uterus is able to undergo a transformation into a similar altered state, in which the blastocysts are capable of transmitting and receiving signals with the uterus in order to facilitate apposition, attachment, and intimate physical and physiological contact with the uterus [1,10,20,24]. Likewise, the understanding of molecular events in the porcine endometrium during pregnancy will also contribute to human clinical translation.

In this study, we aimed to provide a detailed profile of the proteome of the endometrium in pregnancy compared with the non-pregnancy (e NP) state and investigated the changes in the molecular expression profile during pregnancy (40, 70, and 93 days) followed by purification of these proteins. We found 98 proteins regulated differentially among non-pregnant and pregnant tissues (matched and unmatched spots) and identified 63 up- or down-regulated proteins. Interestingly, 10 of these 63 proteins are related to development, cytoskeleton and chaperones, such as transferrin, protein DJ-1, transgelin, galectin-1, septin 2, stathmin 1, cofilin 1, fascin 1, HSP 90β and HSP 27. Our proteomic studies highlight the potential of protein expression profiles for developing molecular diagnoses and for identifying molecular targets for potential therapeutic purposes.

## Results

### 1. Proteomic analysis of endometrial development and Identifications of Up-and Down-regulated Proteins

**Figure 1:**
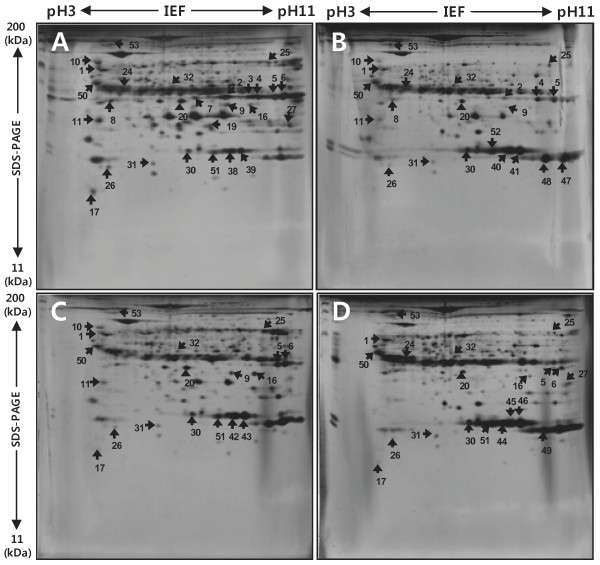
**Representative silver-stained 2D gel images of the different developmental stages of porcine endometrium**. Proteins isolated from e NP, e 40, e 70 and e 93 with 200 μg of total protein loaded into the 2-DE gel. First dimension: 18 cm, with a pH of 3 to 11 linear IPG; second dimension: 12% (A, e NP; B, e 40; C, e 70; D, e 93). Among 3,000 spots visualized using silver staining, 53 proteins were identified from 12% and 8% gels (Figure 1 and 2). Protein spots, which changed by more than twofold compared to the e NP control, were marked and identified using MALDI-TOF and MALDI-TOF/TOF MS.

**Figure 2:**
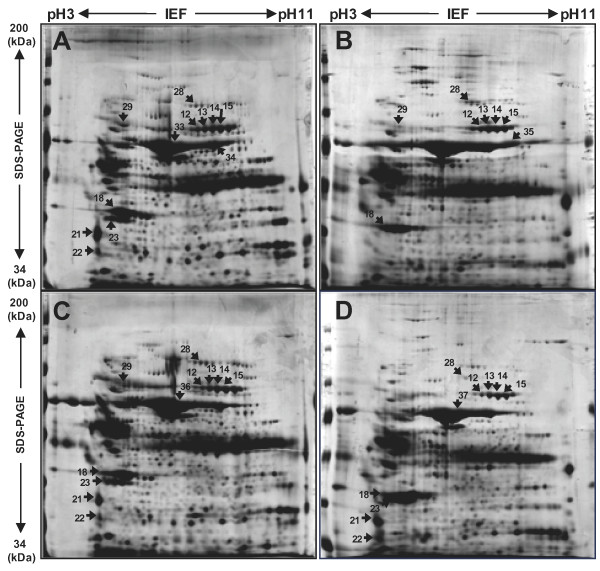
**Representative silver-stained 2D gel images of the different developmental stages of porcine endometrium**. Proteins isolated from e NP, e 40, e 70 and e 93 with 200 μg of total protein loaded into the 2-DE gel. First dimension: 18 cm, with a pH of 3 to 11 linear IPG; second dimension: 8% gels (A, e NP; B, e 40; C, e 70; D, e 93). Among 3,000 spots visualized using silver staining, 53 proteins were identified from 12% and 8% gels (Figure A and B). Protein spots, which changed by more than twofold compared to the e NP control, were marked and identified using MALDI-TOF and MALDI-TOF/TOF MS.

The porcine endometria were examined from maternal tissue at e 40, e 70, and e 93 and from e NP adult pigs. Protein extracts were harvested in lysis buffer and separated by 2-DE using strips with pH 3-11 NL IPG and large format 12% (Figure 1A, B, C and 1D) and 8% SDS gels (Figure 2A, B, C and 2D). 2-DE gels were of high quality in terms of resolution.

Of approximately 1,200 protein spots mapped in the 2-DE gels, we only considered protein spots that showed more than 1.5-fold changes in relative abundance between e NP adult pig and e 40 (p ≤ 0.05). According to these criteria, we excised 53 spots from a single silver nitrate-stained gel for mass spectrometry analysis. Peptide mass fingerprints and peptide-fragment ions were used to identify the spots. The mass accuracy of tryptic peptides was ± 0.05 Da. For peptide sequencing, the number of analyzed peptides and the Mascot scores were specified. Only the Mascot database query results statistically significant at the 5% level were further analyzed (p < 0.05). Mass spectrometry and protein database analysis of these protein spots identified 53 proteins spots. In most cases, the experimental isoelectric points and molecular weights of the identified proteins were in agreement with their theoretical values, as predicted using EXPASY (http://www.expasy.org). Table 1 and Table 2 summarize the differentiation-associated expression change of the up-regulated (1.5-5.3 fold changes in expression levels) and down-regulated (2.5-12.5 fold changes or reductions to almost undetectable levels) proteins, respectively. Several spots were shown to be modified forms or isoforms of nearby protein spots, including transgelin (spot numbers 2, 3, 4, 5, 6 and 7), albumin (spots 33, 34, 35, 36 and 37), transferrin (spots 12, 13, 14, 15 and 16), chain B, structure determination of aquomet porcine hemoglobin At 2.8 Angstrom resolution (spots 38, 39, 40, 42, 42, 43, 44, 45 and 46), hemoglobin subunit alpha (spots 47, 48, and 49) smooth muscle protein (spots 16 and 19), and hemoglobin subunit beta (spots 51 and 52). The identified spots represented 53 unique proteins, of which 29 were up-regulated and 24 were down-regulated. We confirmed that similar protein profiles were repeatedly observed from independent 2-DE experiments (n = 3 in total), which excluded the possibility that the heterogeneous nature of e 40, e 70, and e 93 and e NP adult pig would lead to inconsistent results (data not shown).

**Table 1:** Up-regulation proteins in the endometrium of non-pregnant pigs

Spot **No**.	Protein Name	**NCBI Accession No**.	**SwissProt Accession No**.	Method of ID	Score	Peptides Matched	Peptides Obtained	Sequence Coverage (%)	MW [Da]	PI	MS/Ms Score
1	SEPT2 protein	gi|23274163	Q15019	A	100	13	79	45	42,886	6.38	
2	Transgelin	gi|48255905	Q01995	A	77	9	37	36	22,828	8.01	
3	Transgelin	gi|48255905	Q01995	A	95	9	25	48	22,828	8.01	
4	Transgelin	gi|48255905	Q01995	A, B	90	12	36	54	22,828	8.01	53
5	Transgelin	gi|48255905	Q01995	A, B	72	8	32	44	22,828	8.01	41
6	Transgelin	gi|48255905	Q01995	A	70	5	26	55	22,828	8.01	
7	Transgelin	gi|48255905	Q01995	A	83	10	49	44	22,828	8.01	
8	Protein DJ-1	gi|56404943	Q99497	A	64	7	36	42	20,050	6.33	
9	Cofilin 1	gi|5031635	P23528	A	201	7	65	54	18,719	8.22	
10	Fascin 1	gi|4507115	Q16658	A	198	24	98	51	55,123	6.84	
11	Cellular retinoic acid binding protein 1	gi|4758052	P29762	A, B	105	10	88	67	15,727	5.3	67
12	Transferrin	gi|833800	B3CL06	A	74	15	68	24	78,954	6.76	
13	Transferrin	gi|833800	B3CL06	A, B	89	11	50	19	78,954	6.76	57
14	Transferrin	gi|833800	B3CL06	A	119	15	33	28	78,954	6.76	
15	Transferrin	gi|833800	B3CL06	A, B	118	16	54	27	78,954	6.76	29
16	Smooth muscle protein	gi|177175	P62736	A	81	10	65	54	22,518	8.56	
17	Smooth muscle protein 22-alpha [Fragment]	gi|75038933	O62766	A	68	4	33	70	10,156	4.93	
18	Smooth muscle gamma-actin	gi|950002	P63267	A, B	95	9	48	40	43,251	5.36	108
19	Smooth muscle protein	gi|177175	P62736	A	108	12	59	55	22,518	8.56	
20	Nck-associated protein 5 isoform 1	gi|126362961	O14513	A	70	20	73	14	211,001	7.17	
21	Alpha-actin	gi|49870	P68137	A, B	83	11	64	48	39,465	5.83	118
22	Beta-actin	gi|9864780	P60709	A	75	8	69	42	32,202	5.15	
23	Gamma-actin	gi|809561	P63260	A	75	9	50	44	41,335	5.56	
24	Similar to actin alpha 1 skeletal muscle protein isoform 1	gi|114631697	P68133	A	78	9	63	42	29,335	5.67	
25	Similar to Uncharacterized protein C9orf68	gi|149736845	Q8N4H0	A	70	7	25	22	46,046	9.35	
26	Beta-globin	gi|120564455	P68871	A	82	6	52	42	14,010	6.04	
27	TRIMCyp	gi|76576111	B0ZE27	A	76	8	21	43	18,194	8.26	
28	Similar to actinin, alpha 4 isoform 13	gi|73947742	O43707	A	73	9	32	14	108,550	5.3	
29	Hsp 90 beta	gi|20149594	P08238	A	82	4	13	24	83554	4.97	

Method of ID: A) MALDI-TOF, B) MALDI-TOF/TOF MS.

**Table 2:** Down-regulation proteins in the endometrium of non-pregnant pigs

**SpotNo**.	Protein Name	**NCBI Accession No**.	**SwissProt Accession No**.	Method of ID	Score	Peptides Matched	Peptides Obtained	Sequence Coverage (%)	MW [Da]	PI	MS/Ms Score
30	Galectin-1	gi|47716872	P09382	A, B	61	4	49	32	14,932	5.07	83
31	Stathmin 1/oncoprotein 18	gi|122890671	P16949	A	75	8	69	53	13,597	9.76	
32	Hsp27	gi|75062102	Q5S1U1	A	76	5	18	15	24,871	6.04	
33	Albumin	gi|833798	P02768	A	139	14	32	25	71,362	5.92	
34	Albumin	gi|833798	P02768	A	142	14	32	25	71,362	5.92	
35	Albumin	gi|833798	P02768	A, B	76	10	40	20	71,362	5.92	38
36	Albumin	gi|833798	P02768	A	80	7	16	17	71,362	5.92	
37	Albumin	gi|833798	P02768	A, B	81	11	40	18	71,362	5.92	31
38	Chain B, Structure Determination Of Aquomet Porcine Hemoglobin At 2.8 Angstrom Resolution	gi|809283	P02067	A	92	7	23	59	16,082	6.76	
39	Chain B, Structure Determination Of Aquomet Porcine Hemoglobin At 2.8 Angstrom Resolution	gi|809283	P02067	A	93	8	39	57	16,082	6.76	
40	Chain B, Structure Determination Of Aquomet Porcine Hemoglobin At 2.8 Angstrom Resolution	gi|809283	P02067	A	111	8	32	59	16,082	6.76	
41	Chain B, Structure Determination Of Aquomet Porcine Hemoglobin At 2.8 Angstrom Resolution	gi|809283	P02067	A	102	7	31	59	16,082	6.76	
42	Chain B, Structure Determination Of Aquomet Porcine Hemoglobin At 2.8 Angstrom Resolution	gi|809283	P02067	A	105	6	13	55	16,082	6.76	
43	Chain B, Structure Determination Of Aquomet Porcine Hemoglobin At 2.8 Angstrom Resolution	gi|809283	P02067	A, B	98	7	21	59	16,082	6.76	19
44	Chain B, Structure Determination Of Aquomet Porcine Hemoglobin At 2.8 Angstrom Resolution	gi|809283	P02067	A, B	129	11	66	82	16,082	6.76	40
45	Chain B, Structure Determination Of Aquomet Porcine Hemoglobin At 2.8 Angstrom Resolution	gi|809283	P02067	A	120	9	19	70	16,082	6.76	
46	Chain B, Structure Determination Of Aquomet Porcine Hemoglobin At 2.8 Angstrom Resolution	gi|809283	P02067	A	114	9	55	65	16,082	6.76	
47	Hemoglobin subunit alpha (Hemoglobin alpha chain) (Alpha-globin)	gi|122465	P69905	A	68	6	39	43	15,087	8.76	
48	Hemoglobin subunit alpha (Hemoglobin alpha chain) (Alpha-globin)	gi|122465	P69905	A	102	10	97	53	15,087	8.76	
49	Hemoglobin subunit alpha (Hemoglobin alpha chain) (Alpha-globin)	gi|122465	P69905	A	69	6	35	40	15,087	8.76	
50	Tropomyosin alpha-1 chain	gi|158931149	P42639	A, B	43	4	17	10	32,744	4.71	41
51	Hemoglobin subunit beta	gi|261245058	P02067	A, B	78	8	64	55	16,212	7.1	96
52	Hemoglobin subunit beta	gi|261245058	P02067	A, B	104	8	50	55	16,212	7.1	40
53	Similar to 78 kDa glucose-regulated protein precursor (GRP 78) (Immunoglobulin heavy chain binding protein) (BiP) (Endoplasmic reticulum lumenal Ca(2+) binding protein grp78) isoform 4	gi|73968068	P34935	A	96	11	40	24	66,329	5.06	

Method of ID: A) MALDI-TOF, B) MALDI-TOF/TOF MS.

### 2. Classification of the Regulated Proteins

A total of 53 protein spots were differentially expressed between the control e NP adult pig and embryos examined at e 40, e 70, and e 93.

**Figure 3:**
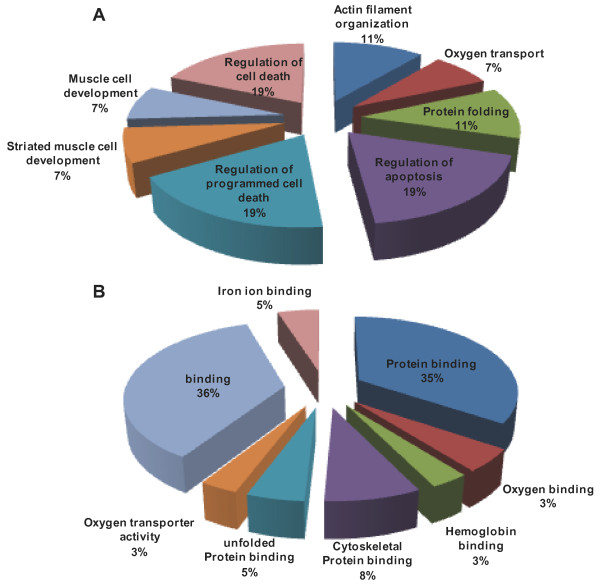
**Classification of differentially regulated proteins from the proteomic analysis**. Ontological classification of differentially regulated proteins in terms of (A) biological process and (B) molecular function using the Gene Ontology (http://www.geneontology.org) and UniProt (http://www.uniprot.org) websites. The compositions of the identified proteins are presented as percentages of all individually identified proteins.

The 53 proteins were assigned to 8 biological processes and 8 molecular functional categories processes according to the information from the Gene Ontology (http://www.geneontology.org) and UniProt (http://www.uniprot.org) websites (Figure 3). The list of proteins was then used to compile a list of associated ontological terms, which are displayed as a histogram. Figure 3 shows that the 53 identified proteins can be functionally classified to 8 categories. Based on the biological process heading, the proteins are predicted to participate in Actin filament organization (11%), Oxygen transport (7%), Protein folding (11%), Regulation of apoptosis (19%), Regulation of programmed cell death (19%), Muscle cell development (7%), and Regulation of cell death (19%) (Figure 3A).

Based on the molecular function headings, the proteins were predicted to be associated with Protein binding (35%), Oxygen binding (3%), Hemoglobin binding (3%), Cytoskeletal Protein binding (8%), unfolded Protein binding (5%), Oxygen transporter activity (3%), and binding (36%) (Figure 3B).

In this study, the binding and apoptosis categories predominated with eighteen and five proteins, respectively.

Among them, we focused on development and cell death related proteins, including transferrin, protein DJ-1, transgelin and galectin 1. HSPs including HSP 90α, HSP 90β, HSP 70, HSP 60 and HSP 27 were also studied because of their chaperoning and anti-apoptotic function. Furthermore, cytoskeleton related proteins including septin 2, stathmin 1, cofilin 1 and fascin1 were investigated for their role in maintaining pregnancy.

### 3. Expression of pregnancy-related proteins in endometrial tissue during the course of pregnancy

We were interested in pathways involved in the development and differentiation of endometrial tissue during the course of pregnancy in pigs, so we focused on the categories related to the structural constituents of cytoskeleton, myoblast differentiation, muscle fiber development and apoptosis.

**Figure 4:**
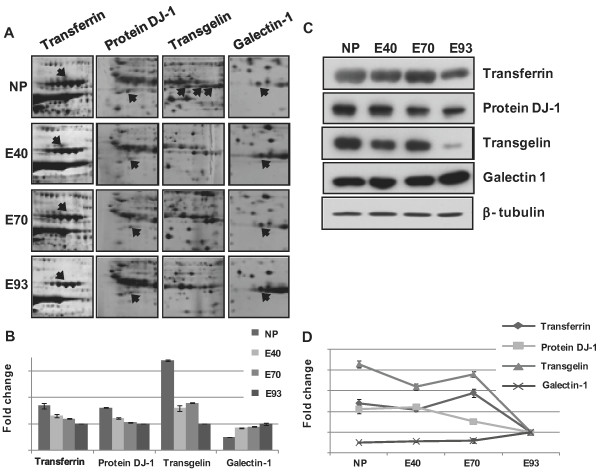
**Enlarged images of differentially expressed protein spots related to development and cell death and confirmation of proteins by western blotting among eNP, e 40, e 70 and e 93. **(A) Different expression of transferrin, protein DJ-1, transgelin and galectin-1 proteins during different developmental stages of porcine endometrium. (B) The amount of protein expression was measured by scanning and the TINA analysis program. (C) Confirmation of differentially expressed proteins in the different developmental stages of porcine endometrium by western blot analysis (transferrin, Protein DJ-1, transgelin and galectin-1 proteins). β-tubulin was used as an internal control. (D) Quantification of PRX1 transferrin, protein DJ-1, transgelin and galectin-1 proteins expression during porcine pregnancy.

Figure 4 shows representative features of protein spots, particularly the development-associated protein spots. The differences in protein expression levels during maintaining pregnancy of the pig endometrial tissue were further verified by western blot analyses.

As expected, the expression of development-associated proteins, such as transferrin, protein DJ-1 and transgelin, by pig endometrial tissue decreased during development and galectin 1 expression by pig endometrial tissue increased during development (Figure 4). In case of transferin, the expressional change was show just a small difference during e NP, e 40 and e 70, but there was expressional change with 2.2 fold change between e NP and e 93. Among these development associated proteins, tarnsgelin, a smooth muscle actin-binding protein with three isoforms, was the most differentially expressed protein with 4.2 fold change between e NP and e 93.

**Figure 5:**
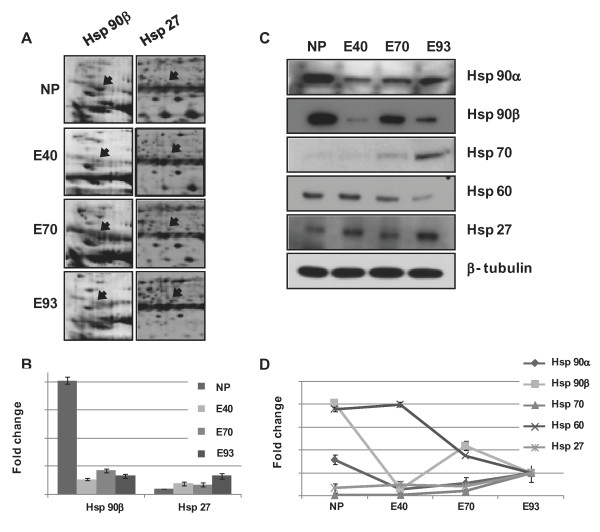
**Enlarged images of differentially expressed protein spots related to chaperoning and anti-apoptotic function and confirmation of proteins by western blotting among e NP, e 40, e 70 and e 93. **(A) Different expression of Hsp 90β and Hsp 27 during different developmental stages of porcine endometrium. (B) The amount of protein expression was measured by scanning and the TINA analysis program. (C) Confirmation of differentially expressed proteins in different developmental stages of porcine endometrium by western blot analysis (Hsp 90α, Hsp 90β, Hsp70, Hsp60 and Hsp27 proteins). β-tubulin was used as an internal control. (D) Quantification of Hsp 90α, Hsp 90β, Hsp70, Hsp60 and Hsp27 protein expression during porcine pregnancy.

Because, in 2-DE analylsis, HSP 90 β and HSP 27 were detected as differentially expressed proteins, we also verified the expression of several HSPs, including HSP 90α, HSP 90β, HSP 70, HSP60 and HSP 27, during pregnancy compared with e NP using western blotting (Figure 5). The expression of several HSP families was not shown regular pattern during development.

The expression of Hsp60 by pig endometrial tissue decreased during development and HSP70 expression by pig endometrial tissue increased during development, gradually. In particular, HSP90α and β were expressed at high concentrations in e NP endometrial tissue and decreased during development compared with e NP approximately with 1.5 and 4.1 fold change, respectively. And HSP27 was abundant in e 93 endometrial tissues and increased with 3.7 fold change compared with e NP (Figure 5).

**Figure 6:**
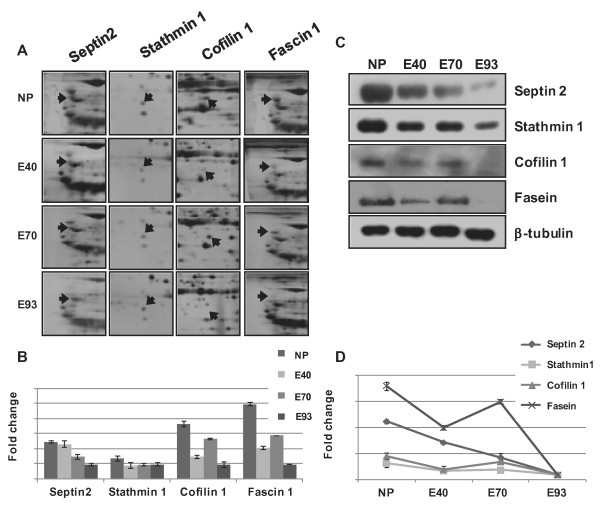
**Enlarged images of differentially expressed cytoskeleton related protein spots and confirmation of proteins by western blotting among e NP, e 40, e 70 and e 93. **(A) Different expression of Septin 2, Stathmin 1, Cofilin 1 and Fascin 1 proteins during different developmental stages of the porcine endometrium. (B) The amount of protein expression was measured by scanning and the TINA analysis program. (C) Confirmation of differentially expressed proteins in the different developmental stages of porcine endometrium by western blot analysis (Septin 2, Stathmin 1, Cofilin 1 and Fascin 1 proteins). β-tubulin was used as an internal control. (D) Quantification of Septin 2, Stathmin 1, Cofilin 1 and Fascin 1 protein expression during porcine pregnancy.

In the Molecular Function category, the expression of cytoskeleton-associated proteins, such as septin 2 and stathmin 1, by pig endometrial tissue decreased with 12.5 and 3.5 fold change, respectively, during development. However, cofilin 1 and fasein 1 were expressed at high concentrations in e NP and e 70 endometrial tissues and decreased during development, compared with e NP, with 4.5 and 18 fold change in e 93 (Figure 6).

## Discussion

The use of animal models to study implantation is of great importance in clinical translation. The advent of 2-DE protein profiling has helped to elucidate global protein changes that allow implantation in pigs, and protein targeting experiments in pigs have resolved the uterine function of many proteins during implantation.

In several studies, significant information has been generated on phase-specific transcriptomes of the endometrium across the menstrual cycle or during different phases of pregnancy. These efforts have tremendously advanced our understanding of endometrial biology. A few studies have been conducted on the comparative protein profiling of the proliferative and secretory phases of the endometrium using 2-DE analysis; however, quantitative or qualitative data on the modulation of the global protein profile of the endometrium during pregnancy compared with the non-pregnancy state are sparse.

In the present study, the protein expression patterns during pregnancy (e 40, e 70 and e 93 day) compared with non-pregnancy state samples were analyzed with quantitative, high resolution 2-DE analysis. This proteomic technique is well suited for the identification of proteins with altered expression during various conditions. The method has wide applications, e.g., for the comparison of protein expression patterns of different cell types, cell differentiation and transformation and in response to various stimuli. We recently used the technique to identify proteins during the pregnancy (e 40, e 70 and e 93) and non-pregnancy states, and characterized several proteins not previously described to exhibit variation during the normal pregnancy condition in addition to proteins previously described to be cell development or cell cytoskeleton. By employing MALDI-TOF/TOF MS combined with sequence database searching, we unambiguously identified 53 of the 98 differentially expressed proteins.

As the result, a total of 53 spots were differentially expressed in the endometrium obtained from non-pregnant and pregnant adult pigs examined at e40, e70, and e93. The 53 proteins were assigned to 9 biological processes and 8 molecular functional categories according to information from the Gene Ontology (http://www.geneontology.org) and UniProt (http://www.uniprot.org) websites. These proteins could be classified according to their functional involvement during pregnancy.

Based on their biological process, the identified differentially expressed proteins are related to the regulation of cell death. According to their molecular function heading, the differentially expressed proteins are involved in binding and function as structural constituents of the cytoskeleton. Maintaining pregnancy is closely related with development, cell differentiation, cell proliferation and cell death. Among the identified proteins, transferrin, protein DJ-1, transgelin and galectin 1 are associated with these functions.

Transferrin functions in blood plasma for iron delivery. Iron-containing proteins catalyze key reactions involved in oxygen sensing, energy metabolism, respiration, folate metabolism, and DNA synthesis. Transferrin is not only an iron-binding protein, but also a factor involved in cell proliferation and differentiation, particularly in muscle differentiation and erythropoiesis [17,25,26].

Protein DJ-1 acts as a positive regulator of androgen receptor-dependent transcription. This protein may also function as a redox-sensitive chaperone, as a sensor for oxidative stress, and apparently protects neurons against oxidative stress and cell death [27,28]. Recently, DJ-1 expressed during zebrafish development and knocked down neuronal cells is more sensitive to programmed cell death [29,30]. These reports suggest that DJ-1 might be involved in a part of the stress-responsive machinery and developmental process.

Transgelin, a smooth muscle actin-binding protein with three isoforms, has not been associated with development, especially in endometriotic lesions. Interestingly, both transgelin have been described as markers for smooth muscle differentiation [24].

Galectin-1 may regulate apoptosis, cell proliferation and cell differentiation. In addition, this protein binds beta-galactoside and a wide array of complex carbohydrates and also inhibits CD45 protein phosphatase activity. Galectins are expressed in the human endometrium and play a role in endometrial regulation. Galectin-1 null mice are deficient in the development of a subset of olfactory neurons and transiently in muscle development [31-34].

Furthermore, several reports have shown that transferrin and galectins-1 are important mediators of inflammation and are major players in the defense against invading micro-organisms. As endometrial function and implantation involve many inflammatory mediators, these proteins might contribute to the modulation of the endometrial immune system. As the endometrial immune system is constantly challenged by bacteria ascending through the cervix, transferrin and galectins-1 might contribute to the protection of the endometrium against bacterial infection [35].

Herein, we also identified and classified HSPs (HSP 90α, HSP 90β, HSP 70, HSP 60 and HSP 27) as differentially expressed proteins during pregnancy. Their chaperone and anti-oxidant functions are correlated with their apoptosis-regulating function against stress *in vivo*.

The HSPs were originally discovered based on their induction in high temperature conditions. They function primarily as molecular chaperones facilitating the folding of other cellular proteins, but also in regulating apoptosis by interacting directly with key components of the apoptotic pathway. Consequently, HSPs are also a highly conserved family of stress response proteins with apoptosis-regulating functions. HSP 90α, HSP 90β, HSP 70, and HSP 27 are anti-oxidants involved in hypoxic conditions and changes in the location of intracellular HSP 60 are associated with apoptosis [36-38].

As steroid hormone synthesis is prominent in the placenta and decidua throughout pregnancy, the abundant expression of HSPs in the endometrium throughout pregnancy is not surprising. In previous reports, striking differences in the cellular and subcellular localization of HSPs 90, 70, 60 and 27 in the decidua and placenta during pregnancy were demonstrated [36]. These differences were concluded to have resulted in part from the different hormonal milieu of cells during pregnancy and different levels of the metabolic activity of cells. Earlier studies by other investigators demonstrated that hormonal regulation of HSP 27 is tissue dependent. For example, HSP 27 levels in the uterine endometrium increase markedly during the luteal phase of the menstrual cycle, whereas levels in the adjacent cervical epithelium do not change [37]. These proteins involved in development, cell differentiation, and cell proliferation also participate in regulating cell death and these functions are correlated with endometrium development and regulation during pregnancy.

Second, maintaining pregnancy is also regulated by cytoskeleton associated proteins. Cell movement is a complex process involving a number of steps, including the disruption of cell-cell junctions, cytoskeletal rearrangements and constant remodeling of adhesive contacts with the ECM. This complexity of events makes evaluation of the entire phenomenon particularly interesting in the context of the endometrium as a dynamic tissue [12,16].

Septins have been suggested to play a role in vesicle trafficking, cytoskeletal remodeling and apoptosis through organizing the actin-myosin ring at that site, regulating the contraction of the ring or the attachment of the actin-myosin ring to the plasma membrane. Alternatively, septins might be involved in the addition of new plasma membranes that presumably must accompany furrow formation by associating with vesicles. Septins were recently proposed to have additional functions in mammals, at sites involving actin dynamics, cell surface organization and vesicle fusion processes [39].

Stathmin 1 is involved in the regulation of the microtubule filament system by destabilizing microtubules by preventing assembly and promoting disassembly of microtubules. Pampfer et al. (1992) first reported that the highest levels of stathmin mRNA expression are detected in the mouse uterus on days 5-7 of pregnancy and in expanded and hatched blastocysts and suggested that stathmin may participate in early mammalian development. Stathmin expression has been shown to increase markedly in the uterus in rats during implantation and decidualization; moreover, stathmin is expressed at higher levels in implantation sites than in uterus segments between implantation sites. Stathmin was localized in the glandular and luminal epithelium, stromal cells and vascular endothelium before implantation of the embryo into the mouse uterus. Interestingly, at the time of implantation and decidualization, stathmin was highly expressed in blood vessels and decidual cells in mice. The period of implantation and decidualization is accompanied by markedly increased vascular permeability and angiogenesis, which are needed for the pregnancy to succeed [40-42].

Cofilin-1 regulates actin dynamics and is targets of many signaling pathways that affect the actin cytoskeleton. The active form, which is dephosphorylated, binds to F-actin and enhances the rate of actin subunit dissociation, and this form is found in the lamellipodia of migrating cells [43].

Fascin 1 is a globular actin cross-linking protein that is essential for cell-to-cell interactions, cellular migration, cell motility and cell-matrix adhesion. The expression of fascin has been studied through the immunohistochemistry of adult human tissues, vascular endothelial cells, neuronal cells, and fibroblast expressed fascin [44].

Endometrial morphology is dynamically regulated by the changing levels of sex steroid hormones during the menstrual cycle or pregnancy. The cyclic architectural modifications observed in the endometrium are achieved through the interplay of a number of cellular events, including the re-structuring of the cytoskeleton. Remodeling of actin fibers is critical for the morphological organization of the cell membrane, for the generation of cell-cell interactions and for cellular adhesion to the extracellular matrix, and all these processes are required for the plasticity of tissues, including the endometrium. The control of cytoplasmic actin is also crucial for regulating endometrium rearrangement during pregnancy [14,35]. Deregulated endometrial cell remodeling, proliferation, adhesion and interaction with the extracellular matrix are important to a variety of endometrial disorders, including dysfunctional uterine bleeding, infertility and endometriosis. In addition, they play a relevant role in endometrial hyperplasia and cancer. Cellular interaction with the extracellular environment is achieved through the development of dynamic remodeling of the actin cytoskeleton, leading to waves of formation and disassembly of focal adhesion sites and changes in the cell membrane morphology. These events eventually lead to the generation of membrane protrusions (such as lamellipodia and filopodia) and traction forces that allow the cells to move.

In the present study, we determined that developmental proteins or cytoskeletal proteins or HSPs were differentially regulated during pregnancy and non-pregnancy, and the expression pattern was maintained or conserved to maintain pregnancy. Our investigation provides novel information on the differential expression of some proteins during pregnancy (e 40, e 70 and e 93 day) compared with the non-pregnancy state. The effects of the differentially regulated proteins on the endometrial cells remain to be elucidated. These investigations will be further discussed about their functional involvement in maintaining pregnancy and may also help in identifying the causes of various endometrial aberrations during pregnancy.

## Conclusion

In summary, we employed a combinatorial proteomics approach using multiple biological replicates, various experimental techniques for protein and peptide fractionation, and several search engines for data mining to characterize the endometrial cell proteome.

This study demonstrates the power of 2-DE analysis to provide a global profile of protein changes in the endometrium, a highly dynamic tissue, as it progresses from non-pregnancy to pregnancy (e 40, e 70 and e 93). We identified differentially regulated proteins not previously known in this tissue as well as important networks that are particularly up- or down-regulated during pregnancy after the endometrium becomes 'receptive' for implantation. The data provide an important framework for understanding the biology of the endometrium in abnormal conditions, including infertility, endometriosis and endometrial cancer.

The finding of the endometrial cell proteome suggested the presence of a wide variety of response proteins that play a vital role in maintaining the pregnancy state.

However, much work still remains to identify factors that are conserved between species. Additionally, variations in the timing and mechanisms of the animal models will help to elucidate the function of these proteins and these models can facilitate the identification of factors necessary in human implantation.

In this study, proteomic analysis provided a general survey of changes in protein expression and represents the first step toward elucidating the definite molecular conditions during pregnancy. These results can be used to compare the protein expression profiling between the pregnancy and non-pregnancy phases and for investigating the maintenance pattern during pregnancy. Thus, these findings can be applied to maintaining the normal pregnancy state.

## Materials and methods

### Animals

In this study, we used a total number of 8 pigs with a mean body weight of 130 ± 10.5 kg. The pigs were killed by an intraarterial injection of pentobarbital (200 mg/kg) after general anesthesia. All procedures in this experiment were approved by the Animal Care and Use Committee of Daegu University and performed in according with animal welfare and ethics.

### Tissues preparation and Protein extraction

Porcine endometrial samples were collected from a local slaughterhouse and transported to the laboratory within 1-1.5 h after exanguination in 0.9% saline supplemented with 100 IU/ml penicillin, 100 μg/ml streptomycin.

Porcine endometrial samples were obtained from maternal tissue at 40 days (e 40, n = 2), 70 days (e 70, n = 2), and 93 days (e 93, n = 2) and from e NP adults (n = 2). In case of e NP group, we collected endometrial samples in 7 days after menstruation. And pregnancy day 0 was calculated by the day when male standing was first observed. Pregnancy was confirmed by recovering conceptuses from the uterine horns. Porcine e 40 day is comparable to mouse e 7.5, e 70 day is comparable to mouse e 12.5 day and porcine e 93 day is comparable to mouse e 17 day. Endometrial cells were dissected and homogenized in 5 mM phosphate buffer (pH 7.0) and Pro-Prep Protein Extraction Solution (iNtRON Biotechnology, Seongnam, Korea). Total protein was prepared according to the manufacturer's instructions (iNtRON Biotechnology, Seongnam, Korea).

### Two-dimensional gel electrophoresis (2-DE)

Isoelectric focusing (IEF) was performed using an IPGphor unit (Amersham, USA) with a pre-cast nonlinear IPG gel strips (18 cm, pH 3-11; Amersham, USA). Three hundred μg of total proteins were mixed with rehydration solution (7 M urea, 2 M thiourea, 4% (w/v) CHAPS, 50 M DTT, and a trace of bromophenol blue) in a final volume of 350 μl and incubated for 12 h at room temperature before separation by IEF at 500 V for 1 h, 1,000 V for 1 h, or 8,000 V for 5 h (50 mA per gel strip). The gel strips were then immediately equilibrated in equilibrium buffer (50 mM Tris-HCl, pH 8.8, 6 M urea, 30% (v/v) glycerol, and 2% (w/v) SDS). Separation in the second dimension was carried out using 10% SDS-PAGE followed by electrophoresis in a Protean II xi 2-D cell (Bio-Rad) at 10 mA for the first 20 min and then at 20 mA until the bromophenol blue reached the bottom of the gel. The procedure was repeated three times for each sample to ensure reproducibility.

### Staining of 2-D gels

The 2-D gels were stained using a Silver Staining Kit (Amersham, USA). Briefly, the gels were fixed in 40% ethanol and 10% acetic acid for 30 min, sensitized in a solution of 25% (w/v) ethanol glutaraldehyde, 5% (w/v) sodium thiosulfate, and 17 g of sodium acetate for 30 min, and washed three times with water for 15 min each. The gels were subsequently immersed in 2.5% (w/v) silver nitrate and 37% (w/v) formaldehyde for 20 min and developed in a mixture of 6.25 g of sodium carbonate and 37% (w/v) formaldehyde for 2-5 min, and the reaction was then stopped in EDTA-Na_2_-2H_2_O.

### Proteomic analysis

Total protein extracts were prepared from pancreas samples using a protein extraction solution (1.0 mM PMSF, 1.0 mM EDTA, 1 M pepstatin A, 1 M leupeptin, and 0.1 M aprotinin). 2-DE was performed using an IPGphor IEF unit as described previously [25] and above. The silver-stained gels were scanned with an ImageScanner (Amersham, USA) and analyzed with Phoretix Expression software (ver. 2005; Nonlinear Dynamics, UK). Destaining and in-gel tryptic digestion of the protein spots were performed as previously described [26]. Xcise (Shimadzu Biotech Co., Japan), an automatic sample preparation system, was used for in-gel digestion, desalting, and plating onto a MALDI-TOF plate. Desalting was performed with ZipTipC^18 ^(Millipore, Bedford, MA, USA), and plating was accomplished using a 4-hydroxy-α-cyano-cinnamic acid (HCCA) matrix solution. The in-gel-digested peptides were analyzed using an ultraflex-TOF/TOF (Bruker Daltonics, Germany) mass spectrometer. The mass spectra were calibrated and processed using Flex Analysis and BioTool 2.2 software (Bruker Daltonics, Germany). Peptide mass fingerprinting (PMF) ion searches was performed using Mascot 2.1 software (http://www.matrixscience.com) integrated with BioTool 2.2. The MSDB 20060831 (3239079 sequences; 1079594700 residues) and NCBInr 20110312 (13366630 sequences; 4577707277 residues) protein databases were searched using the following Mascot settings: taxonomy: Homo sapiens, one incomplete tryptic cleavage allowed, peptide tolerance: 30-50 ppm, fragment tolerance: 0.5 Da, monoisotopic mass, 1+ peptide charge state as HCCA protonation, alkylation of cysteine by carbamidomethylation as a fixed modification, and oxidation of methionine as a variable modification. For each search, statistically significant (p < 0.05) matches were regarded as correct hits.

### MALDI-TOF calibration

The peptides used for the calibration were bradykinin (1-7)_(M+H)+_mono (757.399), angiotensin_ll_(M+H)+_mono (1046.541), angiotensin_1_(M+H)+_mono (1296.684), substance_P_(M+H)+_mono (1347.735), bombesin_(M+H)+_mono (1619.822), renin_substrate_(M+H)+_mono (1758.93), ACTH_clip(1-17)(M+H)+_mono (2093.086), ACTH_clip(18-39)(M+H)+_mono (2465.198), and somatostatin(28) (M+H)+_mono (3147.471).

### Western blot analysis

Proteins from the endometrium were separated by SDS-PAGE, transferred to a nitrocellulose membrane, and incubated in the blocking solution for 2 h at 25°C with 3% TBST (10 mM Tris HCl, pH 7.4, 140 mM NaCl, and 0.1% Tween-20). After incubation with primary and secondary antibodies, the blot was visualized by enhanced chemiluminescence (Amersham Biosciences, Uppsala, Sweden) and the signal intensity of each band was determined by an LAS 3000 instrument (Fuji Photo Film, Tokyo, Japan). The relative protein levels in each sample were normalized against β-actin. The primary polyclonal antibodies used were transferrin (1:2000, Santa Cruz, Delaware, CA), protein DJ-1 (1:1000, Cell Signaling Technology, Beverly, Massachusetts, United States), transgelin (1:2000, Santa Cruz, Delaware, CA), galectin 1 (1:2000, Santa Cruz, Delaware, CA), septin 2 (1:1000, Santa Cruz, Delaware, CA), stathmin 1 (1:1000, Santa Cruz, Delaware, CA), cofilin 1 (1:2000, Santa Cruz, Delaware, CA), fasein (1:1000, Santa Cruz, Delaware, CA), Hsp 90α (1:1000, Santa Cruz, Delaware, CA), Hsp 90β (1:1000, Santa Cruz, Delaware, CA), Hsp 70 (1:1000, Santa Cruz, Delaware, CA), Hsp 60 (1:1000, Santa Cruz, Delaware, CA), Hsp 27 (1:2000, Cell Signaling Technology, Beverly, Massachusetts, United States) and β-actin (1:5000, Chemicon, Temecula, CA).

## Disclosure of Potential Conflicts of interest

The authors declare that they have no competing interests.

## Authors' contributions

JI and J. carried out the conception and design, data analysis and interpretation, and manuscript writing. YJ, DW, SG. and KS. performed western blotting and statistical analysis. YH. KNJ, JS, SK. and DS. participated in its design and coordination and help to collection samples. HM. and DB. participated in conception and design, data analysis and interpretation, financial support and administrative support. All authors read and approved the final manuscript.
